# Preschool teachers provide fewer participation opportunities to working-class students than those from more privileged backgrounds

**DOI:** 10.1073/pnas.2515833122

**Published:** 2025-09-04

**Authors:** Lewis Doyle, Andrei Cimpian, Louise Goupil, Sébastien Goudeau

**Affiliations:** ^a^Centre de Recherches sur la Cognition et l’Apprentissage, Université de Poitiers, CNRS, Poitiers 86073, France; ^b^School of Psychology, University of Surrey, Guildford GU2 7XH, United Kingdom; ^c^Department of Psychology, New York University, New York, NY 10003; ^d^Laboratoire de Psychologie et NeuroCognition, Université Grenoble Alpes, CNRS, Grenoble 38000, France

**Keywords:** inequality, education, social class, teacher bias, interaction

## Abstract

Social class disparities exist from the earliest stages of education. Research has suggested that class-based differences in factors such as socialization practices and access to resources partly explain this phenomenon, but less work has explored whether teachers’ practices also exacerbate these inequalities. Using whole-class observations of 63 preschool classroom discussions (*N* = 226 students, 10 teachers), we coded 7,941 student participation attempts and subsequent responses from teachers. Mixed-effects Bayesian logistic regressions showed that whether students played by the rules by raising their hands or broke the rules by calling out, they were less likely to have their participation attempts accepted if they came from a working-class background, even when their perceived language skills were matched to their middle- and upper-class peers. These results suggest that early schooling experiences may serve to exacerbate inequalities rather than level the playing field.

Education is a significant predictor of life outcomes (1), yet from a young age, social class-based disparities in linguistic and broader educational outcomes begin to emerge (2, 3). Pinpointing why these differences exist so early in schooling is complex and requires an understanding of the interaction between a child’s background and the school context.

It has been argued that education systems tend to place greater value on the experiences, language, and communication strategies of middle- and upper-class students than on those of their working-class peers (4). This may create a cultural mismatch, whereby middle- and upper-class students have more “cultural capital” at their disposal, enabling them to navigate classroom discussions and activities with greater ease and confidence, and subsequently elicit more advantageous attention from their teachers (2, 5). Preschool offers the promise of leveling this playing field (6), and notably, participation in classroom discussions has been shown to have positive effects on children’s outcomes (7). However, children from working-class backgrounds tend to participate significantly less in preschool classroom discussions than their more privileged peers, even when they have the same linguistic ability (2). The existence of such disparities at the earliest stages of schooling raises a critical question: What is the *source* of these social-class disparities? Here, we investigated whether teachers might be partly responsible by showing subtle biases in whose contributions they value.

Teachers are tasked with meeting the educational—and often pastoral—needs of large numbers of children and generally make a big difference to learners’ outcomes (8). However, despite being public officers, teachers—like most humans—are vulnerable to social biases (9). For example, many teachers have an aversion to working in schools that serve low-income communities (10). They also tend to assign lower grades and ability groups to students from lower (vs. higher) social-class backgrounds, even when their work is identical (11). These biases may be more pronounced under high cognitive load, when teachers have less time to control their responses (12), as is the case during face-to-face classroom interactions—the most common form of communication and feedback in preschool. Moreover, research on classroom interactions has highlighted that many teachers’ behaviors are systematically biased by their expectations (13), with students perceived to have greater promise generally receiving more praise, higher-quality interactions, and opportunities for participation during primary and secondary education (14). Similar research has explored classroom-interaction biases in relation to students’ demographic characteristics, such as race (15), but to our knowledge none has focused on how a student’s social class uniquely relates to their teacher’s propensity to provide opportunities for participation during early education. This study therefore aims to investigate social-class bias at what is a particularly vulnerable stage of a child’s learning and development. Given the early divergence of academic trajectories for children from different socioeconomic backgrounds, any evidence of social-class biases in teachers’ practices would indicate that preschool may exacerbate rather than attenuate inequalities.

To test whether early-education teachers’ practices would subtly—but systematically—reinforce social-class disparities, we carried out intensive naturalistic observations of teacher–student interactions in the context of preschool whole-class discussions. We hypothesized that teachers would systematically afford students from middle- and upper-class (vs. working-class) backgrounds more opportunities to participate orally. Teacher-provided opportunities for participation were operationalized through a) invitation responses to students’ raised hands, and b) positive responses to students’ unsolicited participation attempts (e.g., calling out or interrupting). Together, these behaviors provide insight into teachers’ potential social-class biases both when children play by the rules (i.e., raising their hand) and when they break them (i.e., speaking out of turn).

Data were collected by filming whole-class discussions in French preschool classrooms. These observations were intensively coded by researchers—who were blind to the social class of the students—for all instances of raised hands, unsolicited participation, and teachers’ subsequent reactions. We also collected data on students’ social class and perceived language ability.

We analyzed the data with Bayesian mixed-effects logistic regressions and report 95% credible intervals for all log-odds coefficients. These intervals indicate a 0.95 probability that coefficients fall within the reported ranges. Unlike frequentist CI, credible intervals crossing 0 do not rule out genuine effects, as long as 0 falls in the low-probability tail of the posterior distribution.

## Results

### Teachers’ Responses to Raised Hands.

**Fig. 1.**
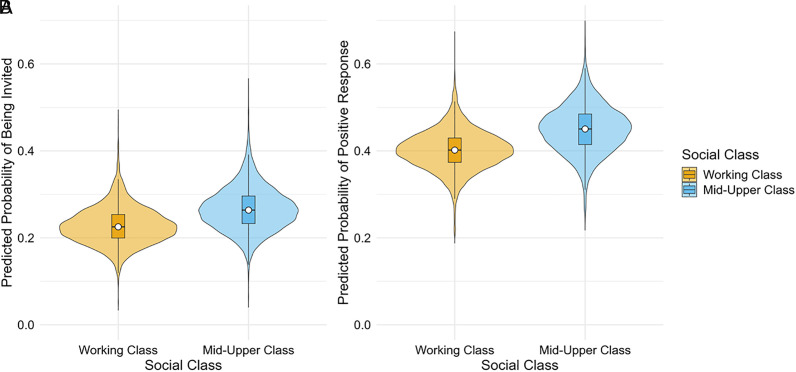
Social-class differences in the model-derived probabilities of (*A*) being invited to speak after raising one’s hand and (*B*) receiving a positive response to unsolicited participation. Boxes indicate interquartile ranges, and white dots signal medians. Estimates control for perceived linguistic ability (*A* and *B*) and hand-raising duration (*A* only).

Students from middle- and upper-class backgrounds were more likely to be invited to participate after raising their hands than those from working-class backgrounds, β = 0.19 [0.01, 0.38]. Importantly, this analysis adjusts for the time that students spent with their hand raised prior to being called on, which means that teachers’ biases cannot simply be due to social-class differences in students’ propensity to volunteer responses or their persistence in doing so. This bias was also observed after additionally adjusting for teachers’ perceptions of students’ language ability, β = 0.21 [0.01, 0.41] (Fig. 1*A*), suggesting that teachers favored the contributions of middle- and upper-class students over those of their working-class peers even when the latter were perceived to have equal linguistic proficiency. This difference equates to an odds ratio of 1.23, indicating that the odds of being invited to participate were 23% higher for students from middle- and upper-class (vs. working-class) backgrounds. There was no independent association between a student’s perceived linguistic ability and their probability of being invited to participate, β = −0.02 [−0.07, 0.03].

### Teachers’ Responses to Unsolicited Participation.

Students from middle- and upper-class backgrounds were more likely to receive a positive response to their unsolicited participation attempts than their peers from working-class backgrounds, β = 0.31 [0.07, 0.55]. This difference was also observed, but was slightly weaker, after adjusting for perceived language ability, β = 0.20 [−0.06, 0.44] (Fig. 1*B*). Thus, teachers valued and encouraged contributions from middle- and upper-class students (vs. linguistically matched working-class students) even when these contributions broke the rules.

Additionally, students with higher perceived linguistic ability (regardless of social-class background) were also more likely to receive a positive response after participating without invitation, β = 0.10 [0.04, 0.16]—even though lower-proficiency students would probably have benefited more from this encouragement.

## Discussion

Our findings suggest that early social-class disparities in education may be compounded by unequal learning opportunities afforded by teachers in the classroom. Based on analyses of 7,941 responses to preschool students’ participation attempts, we found that working-class students were afforded fewer positive responses than their middle- and upper-class peers. Put simply, regardless of whether they played by the rules by raising their hands or broke the rules by calling out or interrupting others in class, working-class children were less likely to be engaged with by their teachers. These findings align with a rich literature on teacher biases (9–14), while also providing insights into how these biases may contribute to early social-class inequalities.

The present findings were broadly robust to controls for perceptions of students’ language proficiency, indicating that something else explained teachers’ biased behaviors. Future research would benefit from measuring whether teachers’ perceptions of other student variables, such as their motivation, ability to stay on topic, or cultural capital, underlie their social-class biases. For example, although lower levels of cultural capital tend to place working-class children at a relative disadvantage in the classroom (2, 5, but see refs. 16 and 17 for counterevidence), this disadvantage may be amplified by teachers’ *perceptions* of their students’ cultural capital. These perceptions may subsequently lead teachers to respond differently to the same behavior (e.g., raising one’s hand) as a function of students’ social class. Indeed, teachers’ interactions often favor students they perceive to be higher-performing (13, 14)—a pattern we also observed in the present research. Teachers’ biases may also be informed by aspects of the school context (18). It would therefore be informative to test, for example, whether the competitiveness of a school’s culture or the level of inequality it shows contribute to the emergence of social-class bias in early education.

In terms of implications, even small differences in teachers’ propensities to enable student participation could, over time, make a substantial difference to the learning opportunities children experience, and ultimately, to their academic outcomes (19). Moreover, young children often interpret lower levels of participation—whether their own or that of their peers—as a sign of individual deficiencies (e.g., intelligence, effort, likeability) (2, 20). Thus, being ignored and rejected during whole-class discussions could also act as a barrier to well-being and development in and outside school. Collectively, these experiences could spark vicious cycles for working-class students, fueled by feelings of rejection, inferiority, and disengagement. By contrast, the comparatively positive responses afforded to middle- and upper-class students could generate feelings of being valued by teachers and peers alike, potentially setting off virtuous cycles.

These findings highlight the need for policymakers, schools, and teacher educators to implement training programs that support teachers to critically reflect on issues of inequality and combat bias (10). Such input could reduce inequalities by increasing all students’ opportunities to participate, learn, and thrive.

## Materials and Methods

This study was approved by the Ethics Committee of the Université Paris Descartes (IRB CER Paris Descartes: 00012019-50). Full details about the methods and materials are available in the *SI Appendix*.

### Participants.

Students (*N* = 226) aged 5 to 6 y and their 10 preschool teachers were filmed across 63 observations. Teachers consented to take part and parents provided consent for their children to participate.

### Data Coding.

Videos were coded for teachers’ responses to students’ a) raised hands (1 = invitation to participate or 0 = no invitation), and b) unsolicited participation (e.g., calling out; 1 = positive response or 0 = anything else). Students’ social class was categorized via their parents’ occupations (*SI Appendix*), and perceived language ability was measured via teacher ratings.

### Analyses.

We specified separate mixed-effects Bayesian binomial logistic regression models to predict the outcome of students’ a) raised hands and b) unsolicited participation from their social class, after adjusting for individual and contextual variables (*SI Appendix*). Posterior estimates (β) are reported on a log-odds scale with 95% credible intervals.

### Open Science.

The coding scheme, data, analytic scripts, and additional analyses are available on the Open Science Framework: https://osf.io/hzf9e/?view_only=2f2ac744cbb943e8b8128fef2b117028 (21).

## Supplementary Material

Appendix 01 (PDF)

## Data Availability

R script, dataset, coding scheme, additional analyses data have been deposited in OSF (https://osf.io/hzf9e/?view_only=2f2ac744cbb943e8b8128fef2b117028) (21).
