# Supplementary material for: Preschool teachers provide fewer participation opportunities to working-class students than those from more privileged backgrounds
Source: Proc Natl Acad Sci U S A. 2025 Sep 4;122(36):e2515833122. doi: 10.1073/pnas.2515833122 (PMC12435286; doi:10.1073/pnas.2515833122)
Supplement: Supplementary file 1 — Appendix 01 (PDF) [file pnas.2515833122.sapp.pdf]

PNAS

Supporting Information for

Preschool teachers provide fewer participation opportunities to working-class students than those from more privileged backgrounds

Lewis Doyle[1,2]*, Andrei Cimpian[3]*, Louise Goupil[4], Sébastien Goudeau[1]

1. Centre de Recherches sur la Cognition et l'Apprentissage, Université de Poitiers, CNRS, 86073 Poitiers, France
2. School of Psychology, University of Surrey, Guildford, GU2 7XH, UK
3. Department of Psychology, New York University, New York, NY 10003, USA
4. Laboratoire de Psychologie et NeuroCognition, Université Grenoble Alpes, CNRS, 38000 Grenoble, France

* Corresponding authors

Email: lewis.doyle@surrey.ac.uk (Lewis Doyle) ; andrei.cimpian@nyu.edu (Andrei Cimpian)

This PDF file includes:

Supporting text
SI References

Other supporting materials for this manuscript include the following:

Dataset (available on the OSF: https://osf.io/hzf9e/?view_only=2f2ac744cbb943e8b8128fef2b117028)

Supporting Information Text

Open Science

The coding scheme, data, R scripts, and additional analyses are available on the Open Science Framework (OSF): https://osf.io/hzf9e/?view_only=2f2ac744cbb943e8b8128fef2b117028. Videos are not available due to restrictions of General Data Protection Regulation rules regarding the anonymity of children.

Participants and Sample Size

The sample consisted of 226 students, aged 5-6 years, and their 10 teachers from six socioeconomically diverse preschools in Western France. Of these, 223 children (117 girls, 106 boys) attempted to participate in some form during the observed sessions. All students were in *Grande Section*, the final year of preschool. We coded approximately 115 minutes of whole-class discussions per teacher (5 to 9 sessions each, depending on session duration), which is broadly in line with previous research on teacher-student interactions (1, 2). Previous research employing similar methods to explore students' participation in class discussions was able to detect small-to-medium effect sizes based on observations of four teachers and 96 students (3). Sensitivity analyses using the *pwr* package in R (4) revealed that our sample was sufficient to detect minimum effect sizes of *OR* = 1.33 with 80% power at the 0.05 alpha level.

Teachers consented to take part and parents provided consent for their children to participate in the study. A full account of the recruitment and video recording procedures is published elsewhere (3).

Materials and Procedure

We carried out intensive naturalistic observations of teacher-student interactions in the context of whole-class discussions, during which students typically attempt to participate either to directly respond to a teacher's question to the class, or to spontaneously add a comment to an ongoing discussion.

Videos were recorded and time stamped using Noldus Media Recorder (Version 2.5, 2013). Four cameras were positioned at different angles in the classroom to enable the coder to see who was speaking at each moment, and footage was then synchronized and aggregated using Noldus Observer XT software. A coding framework was established prior to coding the videos. Using Noldus's The Observer software, 63 video observations, totaling 1,140 minutes ($M_{duration}$ = 18 minutes 6 seconds), were coded by the lead researcher and 20% were double-

coded by one of three research assistants who were blind to the study's hypotheses. Importantly, none of the coders were aware of the social class of the students in the videos. As linguistic cues are often used by perceivers when judging social class (5), we decided to use fluent speakers of the French language who were non-natives (three of whom were from the U.S. and one from the UK) and who therefore had limited knowledge of the subtle class-based differences in accent and dialect, for example, that may be more likely to color the class judgements of someone socialized in the French culture. To test our approach, coders were asked to guess the social class of a random sample of 15 students from their videos. In all cases, binomial tests revealed that these guesses were no better than chance, with an average of 53% correct (ps > .60), thereby giving us confidence that the coding was not biased by coders' perceptions of class. Inter-rater reliability for the coding was $\kappa = .80$, which is close to the threshold of "almost perfect" agreement (6).

Opportunities for Participation

In traditional schooling in Western countries, the two most common ways that students attempt to participate in class discussions are by raising a hand in the hope that they will be invited to speak, and through unsolicited oral participation (for example, calling out to contribute or interrupt), which is generally against the rules. Having one's voice heard in the social learning environment of the preschool classroom may build linguistic and social skills (7, 8) whilst also providing opportunities to practice and act on feedback. Moreover, classroom interactions can signal one's zone of proximal development to the teacher, thereby enabling them to adjust their provision to support learning (9). In contrast, having contributions ignored or reprimanded may have the opposite effects. In all cases, it is typically the teacher who holds the power to accept, shut down, or ignore participation attempts.

Raised hands were coded for their duration and outcome (1 = invitation to participate or 0 = no invitation). Some children raise their hand but become impatient and call out an answer before being invited to speak, so we coded such instances according to the response received from their unsolicited participation: If a student with a raised hand received a positive response after calling out, it was coded as a positive response to unsolicited participation (= 1) and no invitation to participate after raising a hand (= 0), as this would not necessarily have resulted in participation had the student not called out. If the response to their callout was either negative or non-existent, we coded the response to the unsolicited participation as such (= 0) and continued observing the raised hand until it was lowered. In our observations, it was often the case that there were multiple children (sometimes even the majority of the class) with a hand raised at the same time and the teacher had to choose which student to call on. Across the 63 observation sessions, we coded a total of 4,503 instances of hand raising from 214 students.

Unsolicited participation included any instance in which a student called out in class without having been invited to participate. This included interruptions (e.g., the student cuts in over another student or teacher) and non-interruptions (e.g., the student takes the floor spontaneously). Moments where more than 3 students spoke in unison were not coded because in such instances the recipient of teachers' responses was ambiguous. Teachers' responses were coded as positive (e.g., "Yes, excellent point," "Why do you think that?", teacher nodding or smiling) versus other (e.g., "Shhhh!", teacher frowning or shaking head disapprovingly). Both verbal and non-verbal responses were coded. Note that positive responses included responses that disagreed with or corrected students' unsolicited answers but otherwise revealed engagement with the student (e.g., "No, that's not right"), as long as the teacher did not reprimand the student for speaking out of turn. In total, we coded 3,395 instances of unsolicited participation from 173 students. This coding scheme was determined a priori, and informed from both the literature (10-16) and by an initial appraisal of the responses present in our sample.

Social Class

The only indicator of social class available from the French school authorities was parental occupation, which has been shown to be a reliable proxy for overall social class, especially in France (17, 18). We assigned occupations to a social class category (working, middle, or upper) using a classification scheme validated by Goudeau and Croizet (18): Children in the working-class group had parents who were manual and administrative workers, other blue-collar workers (e.g., artisans, farmers), or who were unemployed. Those in the middle-class category had parents in middle-class occupations such as technicians, nurses, or educators. Those in the upper-class group had parents who had the highest ranked roles such as managers, professors, and the professional and managerial elite (e.g., lawyers, doctors). Where occupation information was available for more than one parent, that child's social class classification was based on the parent with the highest-class occupation.

Dichotomization of social class—whereby middle- and upper-class individuals are grouped together and compared with individuals from working-class backgrounds—is common in research in this area (3, 19, 20) and is based on evidence that middle- and upper-class families use socialization and educational practices with young children that are more similar than those adopted in working-class families (21, 22). As such, we ran analyses using the social class data as a binary variable comparing working-class with middle- and upper-class as a combined group.

Due to restrictions on obtaining race- and ethnicity-related information from people in France, we were unable to collect any data pertaining to these demographic variables.

Perceived Language Ability

Teachers rated each student's oral French proficiency level ("How do you estimate the oral proficiency level of this student?") on two different scales (depending on the school). Responses were rescaled onto a common 10-point scale, 1 = *lowest* to 10 = *highest*. This measure was used to determine students' language proficiency because French schools do not administer any formal assessments at this early level of schooling. We argue that adjusting for teachers' ratings of language proficiency in our analyses yields a conservative estimate of bias because these ratings may themselves be biased against working-class students. That is, teachers may underestimate working-class students' ability (23, 24). If we find social class differences in teachers' responses even after adjusting for these (potentially-biased) ratings, it would provide strong evidence for the claim that preschool teachers provide unequal opportunities for participation.

Social class and perceived language ability were significantly correlated, with higher social class predicting higher perceptions of linguistic ability, r = 0.28, p < .001.

Analysis Plan

First, we tested whether the probability of being invited to speak after raising one's hand varied by social class background. We specified a mixed-effects Bayesian binomial logistic regression model using the *brms* package in R (25) with weakly informative Cauchy priors (location = 0, scale = 0.707), indicating that we expected the effect size to be small without ruling out the possibility of a large effect (26). The model predicted the probability of being invited to participate after raising a hand (1 = invitation to participate, 0 = no invitation) from students' social class (− 0.5 = working-class, 0.5 = middle- or upper-class) after controlling for the duration the hand was raised for. The model also included random intercepts for teacher, student, and observation (that is, filming session). We followed this up by running an identical model that also included teachers' perceptions of students' language proficiency as a covariate.

Second, we tested whether the probability of receiving a positive response to unsolicited participation varied according to the student's social class background. We specified a mixed-effects Bayesian binomial logistic regression model—again with weakly informative Cauchy priors (location = 0, scale = 0.707)—with social class predicting the probability of receiving a positive response after unsolicited participation (1 = positive response, 0 = other). Once again, we included in the model random intercepts for teacher, student, and observation. We followed this up by running an identical model that also included language proficiency as a covariate.

The use of Bayesian statistics in psychological research has a range of advantages over more conventional frequentist statistics (27-30). Notably, unlike frequentist statistics, Bayesian statistics afford researchers the ability to calculate the probability that a parameter of interest falls within a certain interval given the existing data. In this study, we report 95% credible intervals, meaning that we can infer a 0.95 probability that our parameters fall within these regions (31). Assuming a normal posterior distribution (as observed; Fig. 1), values that are closer to the center of the credible interval are more probable than values in the tails. This form of analysis places less emphasis on all-or-nothing significance testing, as even credible intervals that cross 0 may signal genuine effects, provided that 0 is in the tail of the posterior distribution (and is thus a low-probability value).

Finally, because our sample size at the teacher level was modest (N = 10), we had limited means of investigating the variability among teachers in the magnitude of their social class biases. Ten clusters (i.e., teachers) are insufficient to reliably estimate the random slope of a variable (a standard measure of the variability across clusters in its relation to the outcome), even with a Bayesian approach (32). However, tentative estimates of inter-teacher variability in social class bias are included in the supplementary results on OSF.

SI References

1. İnan-Kaya, G., & Rubie-Davies, C. M. (2022). Teacher classroom interactions and behaviours: Indications of bias. *Learning and Instruction, 78*, 101516. https://doi.org/10.1016/j.learninstruc.2021.101516
2. Kurkul, K. E., Dwyer, J., & Corriveau, K. H. (2022). 'What do YOU think?': Children's questions, teacher's responses and children's follow-up across diverse preschool settings. *Early Childhood Research Quarterly, 58*, 231–241. https://doi.org/10.1016/j.ecresq.2021.09.010
3. Goudeau, S., Sanrey, C., Autin, F., Stephens, N. M., Markus, H. R., Croizet, J.-C., & Cimpian, A. (2023). Unequal opportunities from the start: Socioeconomic disparities in classroom participation in preschool. *Journal of Experimental Psychology: General, 152*(11), 3135–3152. https://doi.org/10.1037/xge0001437
4. Champely, S. (2020). *pwr: Basic Functions for Power Analysis*. R package version 1.3-0, https://github.com/heliosdrm/pwr
5. Kraus, M. W., Torrez, B., Park, J. W., & Ghayebi, F. (2019). Evidence for the reproduction of social class in brief speech. *Proceedings of the National Academy of Sciences, 116*(46), 22998-23003. https://doi.org/10.1073/pnas.1900500116
6. Landis, J. R., & Koch, G. G. (1977). The measurement of observer agreement for categorical data. *biometrics*, 159-174.
7. Cabell, S. Q., Justice, L. M., McGinty, A. S., DeCoster, J., & Forston, L. D. (2015). Teacher–child conversations in preschool classrooms: Contributions to children's vocabulary development. *Early Childhood Research Quarterly, 30*, 80–92. https://doi.org/10.1016/j.ecresq.2014.09.004
8. Mashburn, A. J., Pianta, R. C., Hamre, B. K., Downer, J. T., Barbarin, O. A., Bryant, D., Burchinal, M., Early, D. M., & Howes, C. (2008). Measures of Classroom Quality in Prekindergarten and Children's Development of Academic, Language, and Social Skills. *Child Development, 79*(3), 732–749. https://doi.org/10.1111/j.1467-8624.2008.01154.x
9. Chaiklin, S. (2003). The zone of proximal development in Vygotsky's analysis of learning and instruction. *Vygotsky's educational theory in cultural context, 1*(2), 39-64.
10. Brophy, J. E., & Good, T. L. (1970). Teachers' communication of differential expectations for children's classroom performance: Some behavioral data. *Journal of Educational Psychology, 61*(5), 365–374. https://doi.org/10.1037/h0029908
11. Brophy, J. E. (1983). Research on the self-fulfilling prophecy and teacher expectations. *Journal of Educational Psychology, 75*(5), 631–661. https://doi.org/10.1037/0022-0663.75.5.631
12. Gentrup, S., Lorenz, G., Kristen, C., & Kogan, I. (2020). Self-fulfilling prophecies in the classroom: Teacher expectations, teacher feedback and student achievement. *Learning and Instruction, 66*, 101296.
13. Harris, M. J., & Rosenthal, R. (1985). Mediation of interpersonal expectancy effects: 31 meta-analyses. *Psychological Bulletin, 97*(3), 363–386. https://doi.org/10.1037/0033-2909.97.3.363
14. Irvine, J. J. (1986). Teacher–student interactions: Effects of student race, sex, and grade level. *Journal of Educational Psychology, 78*(1), 14
15. Pielmeier, M., Huber, S., & Seidel, T. (2018). Is teacher judgment accuracy of students' characteristics beneficial for verbal teacher-student interactions in classroom? *Teaching and Teacher Education, 76*, 255–266. https://doi.org/10.1016/j.tate.2018.01.002
16. Scott, T. M., & Gage, N. (2020). An Examination of the Association Between Teacher's Instructional Practices and School-Wide Disciplinary and Academic Outcomes. *Education and Treatment of Children, 43*(3), 223–235. https://doi.org/10.1007/s43494-020-00024-0
17. Croizet, J.-C., & Claire, T. (1998). Extending the concept of stereotype threat to social class: The intellectual underperformance of students from low socioeconomic

backgrounds. *Personality and Social Psychology Bulletin, 24*(6), 588–594. https://doi.org/10.1177/0146167298246003
18. Goudeau, S., & Croizet, J. C. (2017). Hidden advantages and disadvantages of social class: How classroom settings reproduce social inequality by staging unfair comparison. *Psychological Science, 28*(2), 162–170. https://doi.org/10.1177/0956797616676600
19. Phillips, L. T., Stephens, N. M., Townsend, S. S. M., & Goudeau, S. (2020). Access is not enough: Cultural mismatch persists to limit first-generation students' opportunities for achievement throughout college. *Journal of Personality and Social Psychology, 119*(5), 1112–1131. https://doi.org/ 10.1037/pspi0000234
20. Stephens, N. M., Fryberg, S. A., Markus, H. R., Johnson, C., & Covarrubias, R. (2012). Unseen disadvantage: How American universities' focus on independence undermines the academic performance of first-generation college students. *Journal of Personality and Social Psychology, 102*(6), 1178–1197. https://doi.org/10.1037/a0027143
21. Lahire, B. (2019). *Enfances de classe. De l'inégalité parmi les enfants [Social class childhood. Inequality among children]*. Le Seuil.
22. Lareau, A. (2003). *Unequal childhoods: Class, race, and family life.* University of California Press.
23. Batruch, A., Geven, S., Kessenich, E., & van de Werfhorst, H. G. (2023). Are tracking recommendations biased? A review of teachers' role in the creation of inequalities in tracking decisions. *Teaching and Teacher Education*, *123*, 103985. https://doi.org/10.1016/j.tate.2022.103985
24. Doyle, L., Easterbrook, M. J., & Harris, P. R. (2023). Roles of socioeconomic status, ethnicity and teacher beliefs in academic grading. *British Journal of Educational Psychology*, *93*(1), 91-112. https://doi.org/10.1111/bjep.12541
25. Bürkner, P. (2021). Bayesian Item Response Modeling in R with brms and Stan. *Journal of Statistical Software, 100*(5), 1–54. doi:10.18637/jss.v100.i05.
26. Ly, A., Raj, A., Etz, A., Marsman, M., Gronau, Q. F., & Wagenmakers, E.-J. (2018). Bayesian Reanalyses From Summary Statistics: A Guide for Academic Consumers. *Advances in Methods and Practices in Psychological Science, 1*(3), 367–374. https://doi.org/10.1177/2515245918779348
27. Bian, L., Leslie, S.-J., & Cimpian, A. (2018). Evidence of bias against girls and women in contexts that emphasize intellectual ability. *American Psychologist, 73*(9), 1139–1153. https://doi.org/10.1037/amp0000427
28. Dienes, Z., & Mclatchie, N. (2018). Four reasons to prefer Bayesian analyses over significance testing. *Psychonomic Bulletin & Review, 25*(1), 207–218. https://doi.org/10.3758/s13423-017-1266-z
29. Kruschke, J. K., & Liddell, T. M. (2018). The Bayesian New Statistics: Hypothesis testing, estimation, meta-analysis, and power analysis from a Bayesian perspective. *Psychonomic Bulletin & Review, 25*(1), 178–206. https://doi.org/10.3758/s13423-016-1221-4
30. Van de Schoot, R., Depaoli, S., King, R., Kramer, B., Märtens, K., Tadesse, M. G., ... & Yau, C. (2021). Bayesian statistics and modelling. *Nature Reviews Methods Primers, 1*(1), 1.
31. Morey, R. D., Hoekstra, R., Rouder, J. N., Lee, M. D., & Wagenmakers, E.-J. (2016). The fallacy of placing confidence in confidence intervals. *Psychonomic Bulletin & Review, 23*(1), 103–123. https://doi.org/10.3758/s13423-015-0947-8
32. Stegmueller, D. (2013). How many countries for multilevel modeling? A comparison of frequentist and Bayesian approaches. *American Journal of Political Science, 57*(3), 748-761. https://doi.org/10.1111/ajps.12001
